# Enterocolitis-Associated Pseudo-Obstruction in a Sickle Cell Patient: A Rare Abdominal Catastrophe

**DOI:** 10.7759/cureus.81886

**Published:** 2025-04-08

**Authors:** Munir Ahmad, Mohammed Alblooshi, Abdalla Aboelkheir, Masih Abdul Kader

**Affiliations:** 1 Department of Pediatric Surgery and Urology, Al Jalila Children's Specialty Hospital, Dubai, ARE; 2 Department of Surgery, Tawam Hospital, AI Ain, ARE; 3 Department of Urology, Al Jalila Children’s Specialty Hospital, Dubai, ARE

**Keywords:** colonic pseudo-obstruction, enterocolitis, infectious disease, pediatrics, sickle cell disease

## Abstract

Acute colonic pseudo-obstruction, also known as Ogilvie’s syndrome, is a rare but critical cause of abdominal pain and distension, potentially mimicking mechanical obstruction or toxic megacolon. Patients with sickle cell disease (SCD) have additional risk factors such as vaso-occlusive crises, chronic hemolysis, and susceptibility to infections, which further complicate diagnosis. We report the case of a 14-year-old male patient with SCD who presented with severe generalized abdominal pain, vomiting, and progressive distension with no fever. Imaging revealed significant colonic dilation and pneumatosis, suggesting pseudo-obstruction or ischemic bowel. Infectious evaluations ultimately identified enteropathogenic Escherichia coli. Despite initial concern for toxic megacolon, a multidisciplinary evaluation by hematology, gastroenterology, infectious disease, and surgery confirmed acute colonic pseudo-obstruction associated with enterocolitis. The patient’s condition was managed nonoperatively with nasogastric decompression, intravenous antibiotics, total parenteral nutrition, and prokinetic agents. Serial imaging demonstrated gradual improvement in colonic distension, facilitating a safe return to oral feeding and subsequent discharge. This case underscores the importance of early recognition of enterocolitis-associated colonic pseudo-obstruction in patients with SCD, highlighting the value of comprehensive infection screening and a careful, multidisciplinary management approach to avoid unnecessary surgery and improve outcomes.

## Introduction

Acute abdominal pain in individuals with sickle cell disease (SCD) poses a complex diagnostic challenge. While vaso-occlusive crises remain a leading cause of acute abdominal pain and hospital admission in these patients, other etiologies, including infectious colitis, pseudo-obstruction, and rarely toxic megacolon, must be considered [1]. In particular, intestinal pseudo-obstruction, also referred to as Ogilvie syndrome, is characterized by acute dilation of the colon in the absence of any mechanical obstruction [2]. Although most commonly described in older adults with multiple comorbidities, acute colonic pseudo-obstruction can also occur in pediatric populations, albeit infrequently [3].

Infectious etiologies further complicate the clinical picture. Pathogenic strains of Escherichia coli are increasingly recognized as causes of severe enterocolitis, which can precipitate systemic inflammatory responses and contribute to worsened colonic dilation [4]. When such infections occur in patients with SCD, who already contend with chronic hemolysis, episodic vaso-occlusion, and functional asplenia, the risk of adverse outcomes may be amplified [5]. The combination of an underlying hemoglobinopathy, colonic distension, and potential bowel ischemia or perforation highlights the critical need for an accurate and timely diagnosis.

Despite these risks, the clinical manifestations of SCD can sometimes mask or mimic other conditions. Vaso-occlusive crises may present with abdominal pain that can be difficult to distinguish from an acute surgical abdomen or infectious colitis. Consequently, a structured, multimodal diagnostic approach, including imaging, laboratory investigations, and close multidisciplinary collaboration, is essential to guide appropriate management. This case report details the diagnostic conundrum posed by a 14-year-old male patient with SCD who presented with severe abdominal pain secondary to enterocolitis-associated pseudo-obstruction, ultimately managed successfully without surgical intervention.

## Case presentation

**Figure 1:**
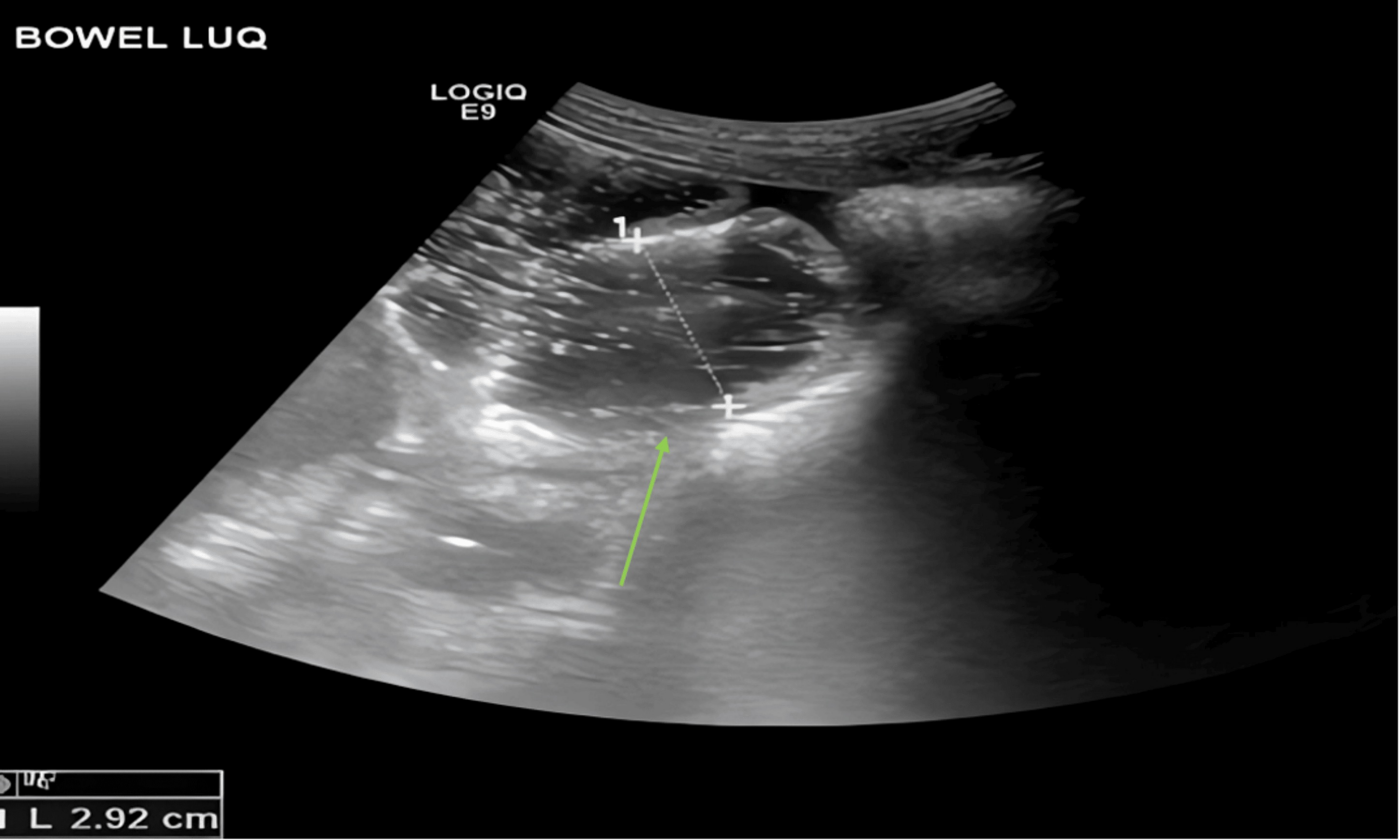
Ultrasound of the Left Upper Quadrant Ultrasound demonstrating markedly distended bowel loops (green arrow) and possible pneumatosis in the left lower quadrant (initial imaging).

**Figure 2:**
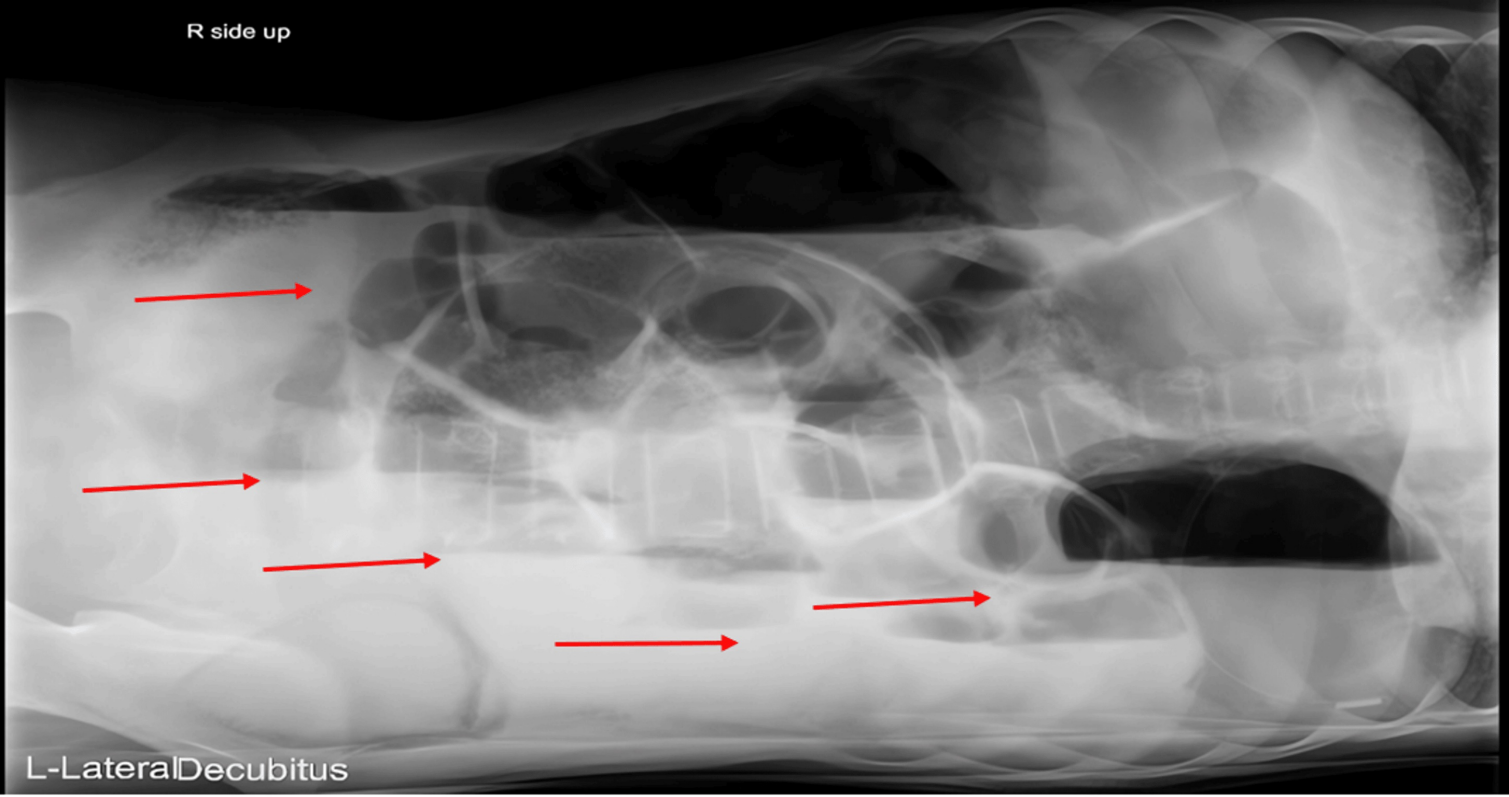
Lateral Decubitus Abdominal Radiograph Initial imaging radiograph revealing significant gaseous distension and multiple air-fluid levels (red arrows).

A 14-year-old boy with known SCD presented to the emergency department with severe, generalized abdominal pain accompanied by abdominal distension and repeated vomiting. He denied fever. Examination revealed diffuse abdominal tenderness without peritoneal signs. An initial bedside ultrasound demonstrated markedly distended bowel loops containing fluid and air, along with a suggestion of pneumatosis in the left lower quadrant (Figure 1). A lateral decubitus abdominal radiograph confirmed significant gaseous distension of both small and large bowel segments, raising concern for an acute abdomen and possible pseudo-obstruction (Figure 2).

Initial laboratory findings and early management

Baseline laboratory testing revealed a white blood cell (WBC) count of 7.2 × 10^9/L (reference range: 4-11 × 10^9/L), hemoglobin of 9.2 g/dL (reference range: 12-16 g/dL), which reflects the patient’s usual baseline rather than an acute drop, given his known SCD, and a mildly elevated reticulocyte count of 120 × 10^9/L (reference range: 25-75 × 10^9/L). C-reactive protein (CRP) was 51 mg/L (reference range: <10 mg/L), and procalcitonin (Pct) was 0.45 ng/mL (reference range: <0.1 ng/mL). An indwelling nasogastric tube (NGT) was placed, yielding 300 mL of dark greenish fluid. The patient was made nil per os (NPO) and started on intravenous fluids and broad-spectrum antibiotics (piperacillin/tazobactam). He was urgently evaluated by hematology to rule out a vaso-occlusive crisis. Although the patient was on hydroxyurea and prophylactic penicillin at home, the hematology consult indicated that he was likely not in an acute sickle crisis. Initial laboratory results and reference ranges are presented in Table 1.

**Table 1:** Summary of Initial Laboratory Findings

Parameter	Value	Reference Range
WBC	7.2 × 10^9/L	4-11 × 10^9/L
Hemoglobin	9.2 g/dL	12-16 g/dL
Reticulocyte Count	120 × 10^9/L	25-75 × 10^9/L
CRP	51 mg/L	<10 mg/L
Procalcitonin (Pct)	0.45 ng/mL	<0.1 ng/mL

Subsequent imaging studies

**Figure 3:**
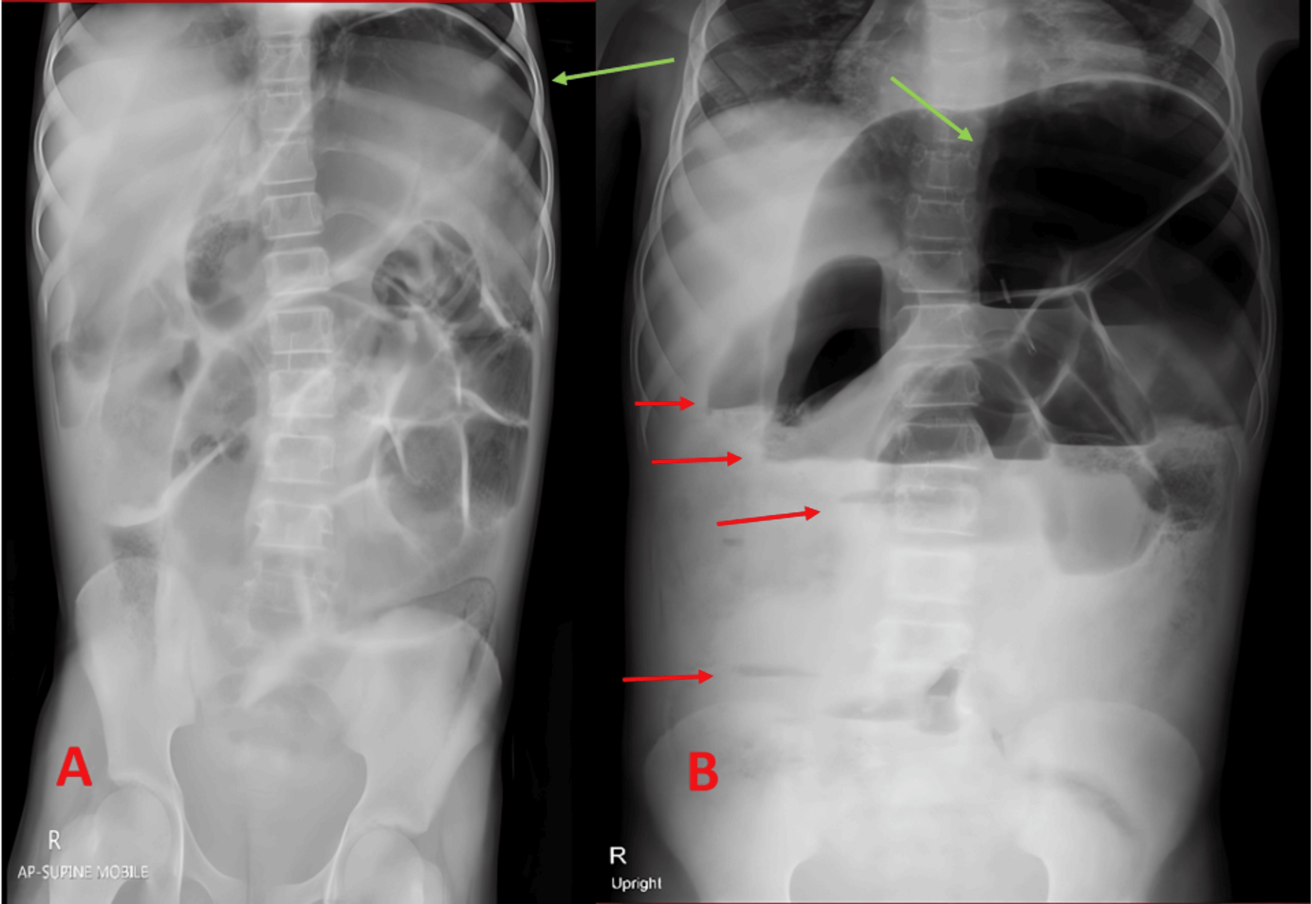
Early Abdominal Radiographs (Acute Distension) (A) Supine AP abdominal radiograph showing widespread dilatation (green arrows) and air-fluid levels (red arrows), (B) Upright abdominal radiograph confirming large air-fluid levels (red arrows) without evidence of a mechanical transition zone.

**Figure 4:**
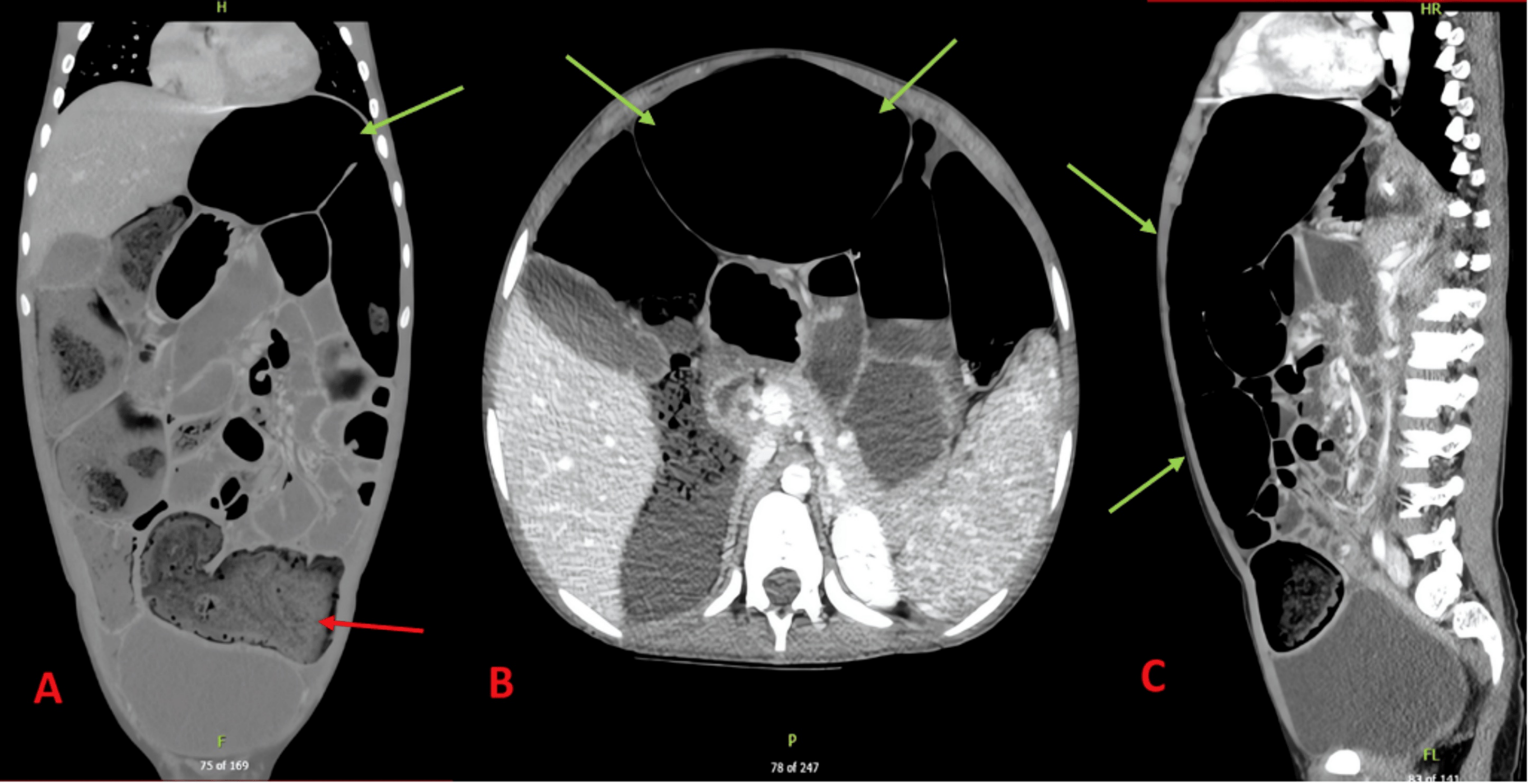
Contrast-Enhanced CT Findings (A) Coronal contrast-enhanced CT showing extensive air- and fluid-filled dilated bowel loops (green arrows). (B) Axial contrast-enhanced CT demonstrating pneumatosis (red arrows) without signs of transmural ischemia. (C) Sagittal contrast-enhanced CT displaying marked colonic distention (green arrows) and bowel wall edema.

Over the next 24 hours, serial plain abdominal radiographs (supine and upright views) showed no evidence of a clear mechanical transition zone but did reveal ongoing and significant distension of the colon and small bowel loops (Figure 3A and Figure 3B). Concern for possible enterocolitis or toxic megacolon prompted a detailed evaluation, including contrast-enhanced computed tomography (CT) of the abdomen and pelvis. The CT scans demonstrated extensive dilation of the colon (maximum transverse colon diameter of approximately 9.7 cm) and pneumatosis in portions of the colon, but no signs of full-thickness ischemia or perforation (Figures 4A-4C). Laboratory values now showed a hemoglobin of 8.2 g/dL (reference range: 12-16 g/dL), WBC of 7.2 × 10^9/L (reference range: 4-11 × 10^9/L), and elevated CRP of 99.1 mg/L (reference range: <10 mg/L). Infectious workup, including stool culture and viral panels, was initiated. Enteropathogenic Escherichia coli was ultimately identified.

Clinical progression and management

On the second hospital day, the patient developed a low-grade fever (38.4 °C), with an increased WBC of 11.1 × 10^9/L and further drop in hemoglobin to 7 g/dL, necessitating packed red blood cell transfusion. A peripherally inserted central catheter (PICC) line was placed for total parenteral nutrition. Additional antibiotics (metronidazole and later vancomycin) were added to cover possible polymicrobial infections and treat a concurrent right lower lobe consolidation noted on chest auscultation.

**Figure 5:**
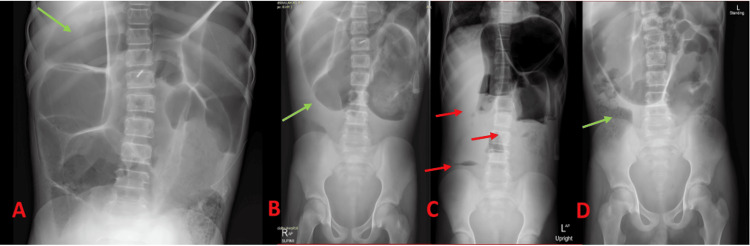
Serial X-rays Demonstrating Clinical Improvement (A) Day 3 supine abdominal radiograph showing persistent colonic distension (green arrows) and air-fluid levels (red arrows). (B) Day 6 supine and upright radiographs (side by side) indicating reduced distension (green arrows) and diminished air-fluid levels (red arrows). (C) Day 11 upright abdominal radiograph confirming near-complete resolution of colonic dilation. (D) Day 11 supine abdominal radiograph showing near-complete resolution from a different view, illustrating continued improvement in colonic distension.

Repeat abdominal radiographs taken on day 3 and day 6 showed gradually improving but persistent colonic distension and air-fluid levels, along with continued absence of a mechanical transition point (Figure 5A and Figure 5B). By day 6, the abdomen was softer, and the patient reported significantly decreased pain. Nasogastric output had also diminished to lighter green fluid totaling 450 mL per day. The patient began passing stool more consistently, and his abdominal distension was noticeably reduced.

Further consultation and recovery

Gastroenterology was consulted for possible neostigmine therapy; however, they favored a conservative approach with prokinetics (erythromycin) rather than pharmacological decompression. Serial imaging continued to show gradual decompression of the colon. By day 8, the NGT drainage was minimal, and it was safely removed. The patient was started on a clear liquid diet, which he tolerated well, and was advanced to a regular diet over the following three days.

**Figure 6:**
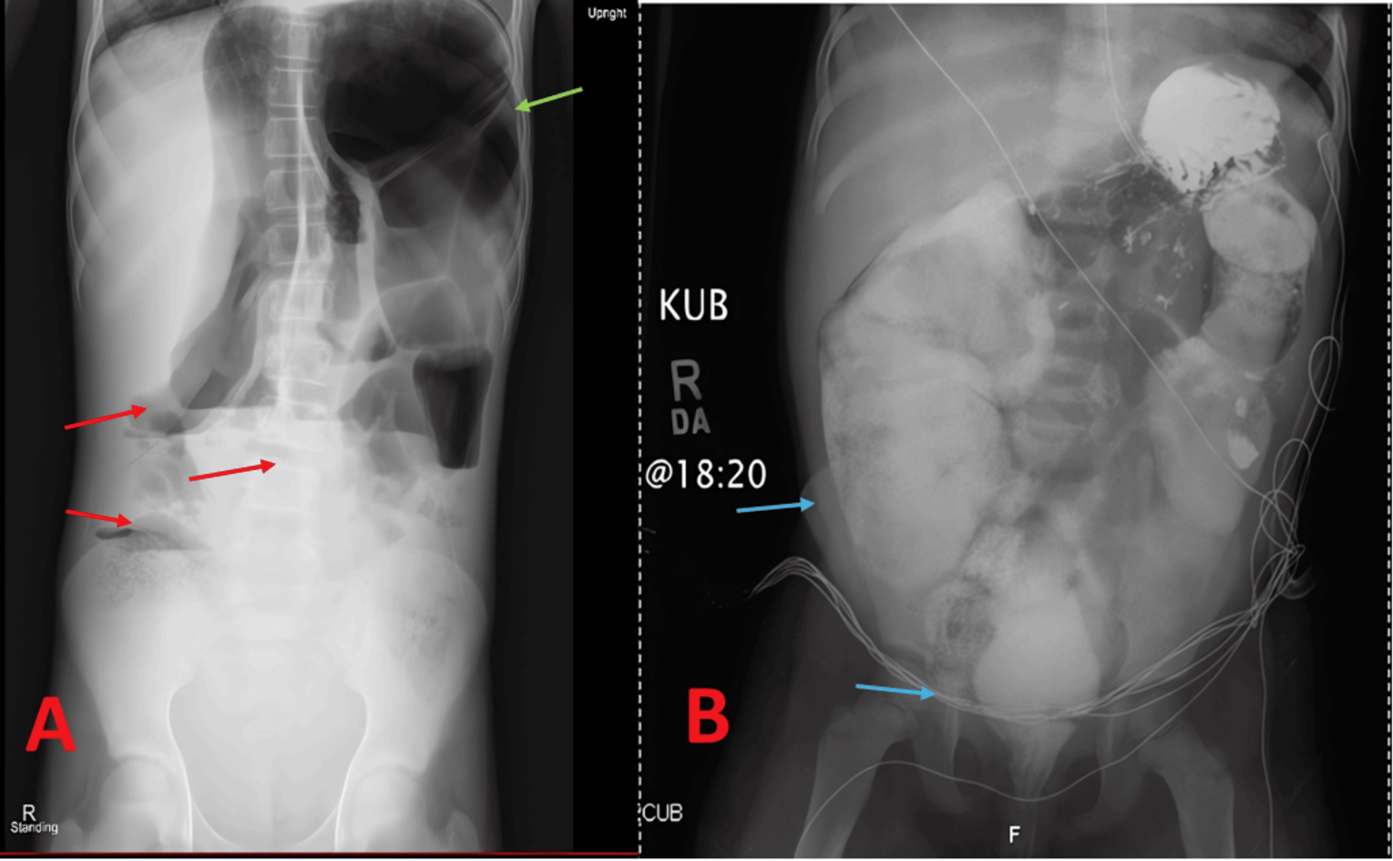
Final Follow-up and Recovery (A) Day 14 upright abdominal radiograph revealing normalization of the bowel gas pattern. Green arrows highlight mildly dilated bowel loops, and red arrows indicate air-fluid levels. (B) Abdominal KUB with lines (blue arrow) demonstrating contrast passing fully through the bowel and reaching the rectum, confirming patency and absence of mechanical obstruction. Minimal residual distension is observed, and the supportive lines remain in place prior to removal.

On day 11, abdominal radiographs displayed near-complete resolution of the colonic distention (Figure 5C and Figure 5D). Follow-up imaging on day 14 confirmed a normal bowel gas pattern (Figure 6). The patient demonstrated full tolerance of a regular diet, negative cultures, and normalization of inflammatory markers. His abdominal exam was benign, and oral intake was fully restored. He was discharged in stable condition, having made a full recovery from enterocolitis-associated acute pseudo-obstruction, with instructions for close follow-up with hematology and gastroenterology.

By day 14, the patient’s abdominal exam was benign, oral intake was fully restored, and all repeat cultures were negative. He was discharged home with close follow-up, having made a full recovery from enterocolitis-associated acute pseudo-obstruction.

To provide a clear overview of the patient’s clinical and laboratory progression, we have summarized key parameters in a timeline format (Table 2). This table highlights how serial imaging and lab results guided our management decisions.

**Table 2:** Timeline of Key Laboratory and Imaging Findings WBC: White blood cell count; CRP: C-reactive protein; RBC: Red blood cells; WNL: Within normal limits; N/R: Not reported

Day	Day 0 (Admission)	Day 1	Day 2	Day 14 (Discharge)
WBC (4-11 × 10^9/L)	7.2 × 10^9/L	7.2 × 10^9/L	11.1 × 10^9/L	Normal / WNL
Hemoglobin (12-16 g/dL)	9.2 g/dL	8.2 g/dL	7.0 g/dL (Required RBC transfusion)	Normal / WNL
Reticulocyte Count (25-75 × 10^9/L)	120 × 10^9/L	N/R	N/R	N/R
CRP (<10 mg/L)	51 mg/L	99.1 mg/L	N/R	Normalized
Procalcitonin (Pct) (<0.1 ng/mL)	0.45 ng/mL	N/R	N/R	N/R
Temperature	Normothermic (~37.0 °C)	Normothermic	38.4 °C (fever)	Afebrile
Key Imaging	X-ray: Significant colonic dilation, possible pneumatosis	CT: Maximum colonic diameter ~9.7 cm; no full-thickness ischemia	X-ray: Persistent colonic dilation	X-ray: Near-complete resolution; normal bowel gas pattern
Additional Notes	IV fluids, nasogastric decompression, broad-spectrum antibiotics (piperacillin/tazobactam)	Stool culture positive for Enteropathogenic E. coli (EPEC)	Started total parenteral nutrition (TPN), added antibiotics (metronidazole ± vancomycin)	Negative cultures; inflammatory markers normalized; stable condition

## Discussion

Acute colonic pseudo-obstruction (ACPO), also known as Ogilvie’s syndrome, is characterized by acute, non-obstructive dilation of the colon in the absence of any mechanical blockage. Though more common in older adults with multiple comorbidities, ACPO has an overall incidence estimated at 0.06% to 0.1% of hospital admissions in adult populations, with mortality rates as high as 10-15% if not promptly recognized and managed [6,7]. While precise incidence data in younger patients remain limited, emerging case reports and small series indicate that it is being increasingly recognized in pediatric and adolescent populations, particularly those with underlying conditions like SCD. In the present case, the patient’s SCD, combined with enteropathogenic Escherichia coli infection, may have precipitated the development of pseudo‐obstruction by inducing localized inflammation, impairing bowel motility, and increasing susceptibility to intestinal dysbiosis.

In SCD, vaso‐occlusive crises and hemolysis contribute to a state of chronic end‐organ dysfunction, including possible alterations in splanchnic circulation. This can lead to heightened intestinal wall vulnerability and slowed transit time under stress. In such a proinflammatory environment, even a modestly virulent organism can initiate a robust mucosal inflammatory response, increasing the risk of bowel dilation and pneumatosis. Moreover, patients with SCD often receive multiple blood transfusions, iron chelation, and prophylactic antibiotics, all of which can shift the gut microbiome and decrease colonization resistance, thereby facilitating opportunistic infections. Gastrointestinal complications in SCD have been well documented, highlighting the role of chronic inflammation and altered gut motility [8].

Distinguishing ACPO from toxic megacolon and mechanical obstruction is a critical diagnostic challenge. Toxic megacolon typically involves a systemic toxicity component and elevated inflammatory markers, often with an underlying inflammatory bowel disease or severe infectious process. In our case, the absence of marked systemic toxicity and the lack of a discrete transition point on imaging supported the diagnosis of pseudo‐obstruction rather than a fulminant colonic inflammatory process. Computed tomography scans showing extensive colonic dilatation and pneumatosis, yet without transmural ischemia, were crucial for diagnosis. These findings, along with stable lactate levels and relatively preserved hemodynamics, supported a conservative management strategy.

Therapeutic considerations in ACPO include bowel decompression via nasogastric or rectal tubes, prokinetics such as erythromycin, and in some instances, neostigmine. In fact, neostigmine has been shown to be effective in reversing colonic distension in patients with ACPO [9]. However, patients with significant cardiac or respiratory comorbidities, or those at high risk for perforation, may require more cautious approaches. Endoscopic decompression and surgery are generally reserved for refractory cases or when there is evidence of perforation or ischemia. In this patient, the progressive reduction in abdominal distension and pneumatosis over several days validated a conservative approach. Identification and management of enteropathogenic Escherichia coli infection and close hemodynamic monitoring further contributed to a favorable clinical course.

Ultimately, this case highlights the importance of multidisciplinary collaboration, integrating hematology, infectious disease, gastroenterology, and surgery, to properly diagnose and manage acute abdominal presentations in patients with SCD. Timely imaging, microbiological investigations, and a watchful, methodical treatment strategy averted unnecessary surgical intervention and allowed for complete clinical recovery. A multidisciplinary approach, incorporating advanced imaging and careful clinical monitoring, is essential in managing ACPO [10].

## Conclusions

In this patient with SCD presenting with acute abdomen, a meticulous diagnostic approach highlighted the interplay of enterocolitis and colonic pseudo-obstruction, ultimately enabling successful nonoperative management. Awareness of pseudo-obstruction in patients with underlying hemoglobinopathies emphasizes the necessity of early, targeted evaluation, close multidisciplinary collaboration, and thoughtful therapeutic decision-making to prevent complications and optimize recovery.
